# Herpes Simplex Virus 1 Glycoproteins Differentially Regulate the Activity of Costimulatory Molecules and T Cells

**DOI:** 10.1128/msphere.00382-22

**Published:** 2022-09-12

**Authors:** Harry H. Matundan, Ujjaldeep Jaggi, Homayon Ghiasi

**Affiliations:** a Center for Neurobiology and Vaccine Development, Ophthalmology Research, Department of Surgery, Cedars-Sinai Burns & Allen Research Institute, CSMC – SSB3, Los Angeles, California, USA; University of Michigan-Ann Arbor

**Keywords:** glycoproteins, HSV-1, plasmids, transfection, promoter analysis, Luminex, cytokines-chemokines, CD4, CD8, CD80, CD86

## Abstract

Over the past 70 years, multiple approaches to develop a prophylactic or therapeutic vaccine to control herpes simplex virus (HSV) infection have failed to protect against primary infection, reactivation, or reinfection. In contrast to many RNA viruses, neither primary HSV infection nor repeated clinical recurrence elicits immune responses capable of completely preventing virus reactivation; yet the 12 known HSV-1 glycoproteins are the major inducers and targets of humoral and cell-mediated immune responses following infection. While costimulatory molecules and CD4/CD8 T cells both contribute significantly to HSV-1-induced immune responses, the specific effects of individual HSV-1 glycoproteins on CD4, CD8, CD80, and CD86 activities are not known. To determine how nine major HSV-1 glycoproteins affect T cells and costimulatory molecule function, we tested the independent effects of gB, gC, gD, gE, gG, gH, gI, gK, and gL on CD4, CD8, CD80, and CD86 promoter activities *in vitro*. gD, gK, and gL had a suppressive effect on CD4, CD8, CD80, and CD86 promoter activities, while gG and gH specifically suppressed CD4 promoter activity. In contrast, gB, gC, gE, and gI stimulated CD4, CD8, CD80, and CD86 promoter activities. Luminex analysis of splenocytes and bone-marrow-derived dendritic cells (BMDCs) transfected with each glycoprotein showed differing cytokine/chemokine milieus with higher responses in splenocytes than in BMDCs. Our results with the tested major HSV-1 glycoproteins suggest that costimulatory molecules and T cell responses to the nine glycoproteins can be divided into (i) stimulators (i.e., gB, gC, gE, and gI), and (ii) nonstimulators (i.e., gD, gK, and gL). Thus, consistent with our previous studies, a cocktail of select HSV-1 viral genes may induce a wider spectrum of immune responses, and thus protection, than individual genes.

**IMPORTANCE** Currently no effective vaccine is available against herpes simplex virus (HSV) infection. Thus, there is a critical need to develop a safe and effective vaccine to prevent and control HSV infection. The development of such approaches will require an advanced understanding of viral genes. This study provides new evidence supporting an approach to maximize vaccine efficacy by using a combination of HSV genes to control HSV infection.

## INTRODUCTION

Of the more than 80 herpes simplex virus 1 (HSV-1) genes, 12 encode proteins that are modified by the addition of host cell carbohydrates (1–9). These genes are envelope proteins and play critical roles in virus attachment, penetration, envelopment, egress, and membrane fusion (10, 11). Some of these glycoproteins (gB, gC, gD, gE, gG, gH, gI, gJ, gK, gL, gM, and gN) are also major inducers and targets of humoral- and cell-mediated immune responses following HSV-1 infection (1–3, 12–14). Glycoproteins induce specific immune response patterns in mice, including T cell cytokine profiles and CD4^+^ versus CD8^+^ coreceptor usage (1–3, 12, 15). The activation and regulation of T cells are the important aspects of adaptive immunity (16). T cell proliferation, differentiation, and cytokine secretion depend on the binding of CD28 on T cells to CD80 or CD86 molecules (17), which are expressed on the surface of multiple cell types, including B cells, macrophages, dendritic cells (DCs), and T cells (18–21). We and others have shown that dendritic cells express high levels of CD80 and CD86 costimulatory molecules that provide the signal required for T cell activation and proliferation (22, 23).

We have reported previously that the binding of HSV-1 ICP22 to the CD80 promoter reduces T cell activation and function, thereby protecting mice infected with HSV-1 (24). We have also shown that the absence of ICP22, using ICP22 null virus, leads to reduced primary virus replication in the eye and enhanced immune cell infiltration, but there was no difference in eye disease in ICP22-null-infected and wild-type (WT)-virus-infected mice (25). We also showed that infection with a KOS-ICP22Δ40 mutant virus resulted in higher levels of virus replication in the eye than seen after infection with ICP22 null virus and enhanced CD80 expression in dendritic cells (22). Overexpression of CD80 by HSV-1 exacerbated eye disease in infected mice and increased CD8^+^ T cells in the corneas of infected mice (22, 24, 25).

Previously, we constructed baculovirus recombinant viruses expressing 11 of HSV-1 glycoproteins and characterized them *in vitro* and *in vivo* (1–4, 12, 26–34). We have shown that (i) immunization with gB, gC, gD, gE, or gI completely protects mice against lethal challenge (26–30); however, eye disease and latency establishment were not eliminated (2, 12). (ii) Immunization with any of the other six glycoproteins did not protect against lethal challenge (1, 3, 4, 12, 31–34). (iii) A cocktail of seven glycoproteins (7gP), consisting of the first seven glycoproteins that were recombinantly expressed in our lab (gB, gC, gD, gE, gG, gH, and gI), provided more efficacious protection than any of these individual glycoproteins (2, 12, 35) and was more efficacious than Chiron’s choice of gB+gD (36). Results suggest that the effectiveness of these seven glycoproteins in protecting against eye disease can be ranked as follows: gD > gB > gI > (gC = gE) > gG > gH (12). (iv) Immunization with 5gP (consisting of the 7gP without the potentially harmful gG and the ineffective gH) was more efficacious than immunization with 7gP (9).

Since costimulatory molecules CD80 and CD86 strengthen T cell activation and function (23), we extended our investigation further into the functional behavior of CD80 and CD86 in viral glycoprotein interactions. Here, we examined the effects of nine individual major viral glycoproteins on CD80 and CD86 promoter activities because CD80 and CD86 have functional roles in CD4 and CD8 T cell responses as well as the effect of these nine viral glycoproteins on CD4 and CD8 promoter activities. Our results showed that the gD, gK, and gL genes had a suppressive effect on CD80, CD86, and CD8 promoter activities, whereas gB, gC, gE, gG, gH, and gI upregulated CD80 and CD86 activities. However, CD4 promoter activities were negatively affected by gD, gK, and gL genes as well as by gG and gH. Transfected splenocytes and bone-marrow-derived dendritic cells (BMDCs) responded differently to each glycoprotein than their infected counterpart. Thus, our results suggest that a proper cocktail of various glycoproteins rather than individual or other combinations of them may provide more effective and long-lasting protection against HSV-1 infection *in vivo*.

## RESULTS

### Effect of HSV-1 glycoproteins on CD80, CD86, CD4, and CD8 promoter activities.

A subset of HSV-1 glycoproteins has been shown to play a major role as inducers and targets of humoral- and cell-mediated immune responses following HSV-1 infection. Therefore, to determine how the HSV-1 gB, gC, gD, gE, gG, gH, gI, gK, and gL glycoproteins affect the promoter activity of CD4, CD8, CD80, and CD86 *in vitro*, we cloned the complete open reading frames (ORFs) of their genes into a pVR-1055 backbone as we described previously (9). The gB, gC, gD, gE, gG, gH, gI, gK, and gL ORFs were described previously (3, 4, 12, 26–32). We also cloned the promoters of CD4, CD8, CD80, and CD86 into the pGL plasmid to create pGL4-CD4p, pGL4-CD8p pGL4-CD80p, pGL4-CD86p, and pGL4-empty vector (EV); pGL4-EV was used as a control as we described previously (24). Sequences of the CD4, CD8, CD80, and CD86 promoters are shown in Table S1 in the supplemental material.

10.1128/msphere.00382-22.1 TABLE S1 CD80, CD86, CD4, and CD8 promoter sequences used in this study. Shows the promoter regions of CD80, CD86, CD4, and CD8 that were synthesized and inserted into the XhoI/SacI site of plasmid pGL4 for CD80 or into BamHI site of plasmid pGL4 for CD86, CD4, or CD8 as described in Materials and Methods. Numbers in parenthesis indicate the start and end of each promoter. Download Table S1, DOCX file, 0.03 MB. Copyright © 2022 Matundan et al. 2022 Matundan et al. https://creativecommons.org/licenses/by/4.0/ This content is distributed under the terms of the Creative Commons Attribution 4.0 International license.

**FIG 1:**
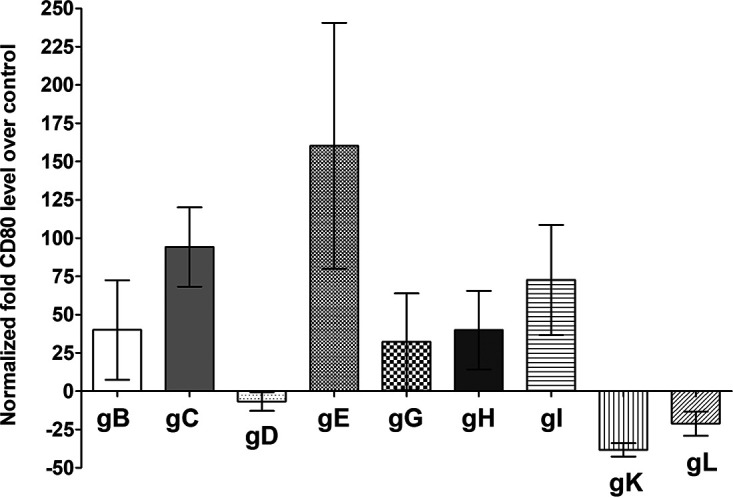
Effects of HSV-1 glycoproteins on CD80 promoter activity. 293 cells were transfected with either pGL4-EV or pGL4-CD80p DNA and then were transfected individually with gB, gC, gD, gE, gG, gH, gI, gK, or gL plasmid DNAs. The effect of each glycoprotein on CD80 promoter activity was determined 48 h posttransfection as we described in Materials and Methods. Assays were conducted in replicates of 10, and means ± SEM were calculated from 3 separate experiments (*n* = 30) for each point. gE is significantly upregulated compared with gL (*P* < 0.05), and no significant differences were detected among other glycoproteins (*P* > 0.05). All *P* values were determined using ANOVA statistical analyses.

We transfected 293 cells with pGL4-CD80p or pGL4-EV DNA and then individually transfected them with gB, gC, gD, gE, gG, gH, gI, gK, and gL for 48 h. An analysis of luciferase promoter activity showed that CD80 expression was significantly enhanced by 150-fold in the gE-transfected group (Fig. 1) (*P* < 0.05). Other glycoproteins, including gB, gC, gG, gH, and gI, also increased CD80 expression but to a lesser extent. CD80 expression was downregulated in gD-, gK-, and gL-transfected cells, (Fig. 1) (*P* > 0.05).

**FIG 2:**
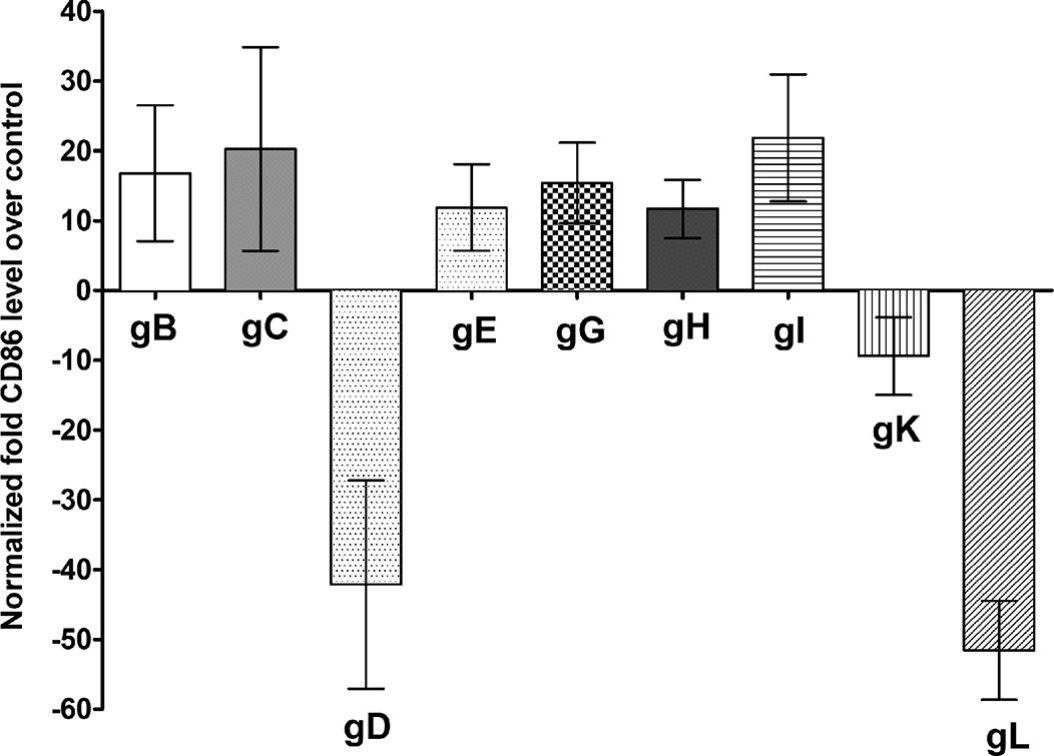
Effects of HSV-1 glycoproteins on CD86 promoter activity. 293 cells were transfected with either pGL4-EV or pGL4-CD86p DNA and then were transfected individually with gB, gC, gD, gE, gG, gH, gI, gK, or gL plasmid DNAs. The effect of each glycoprotein on CD86 promoter activity was determined 48 h posttransfection as we described in Materials and Methods. Assays were conducted in replicates of 10, and means ± SEM were calculated from 3 separate experiments (*n* = 30) for each point. gB is significantly upregulated compared with gD and gL (*P* < 0.004); gC is significantly upregulated compared with gD and gL (*P* < 0.001); gD is significantly downregulated compared with gE, gG, gH, and gI (*P* < 0.002); gE is significantly upregulated compared with gL (*P* = 0.0003); gG is significantly upregulated compared with gL (*P* < 0.0001); gH is significantly upregulated compared with gL (*P* = 0.0001); and gI is significantly upregulated compared with gL (*P* < 0.0001). All *P* values were determined using ANOVA statistical analyses.

To determine the effects of gB, gC, gD, gE, gG, gH, gI, gK, and gL on CD86 promoter activity, we transfected 293 cells with pGL4-CD86p or pGL4-EV DNA followed by transfection with each individual glycoprotein as described above for Fig. 1. The CD86 promoter luciferase activity after cotransfection with each glycoprotein was determined 48 h posttransfection. We found that CD86 expression was significantly downregulated in cells transfected with gD (Fig. 2) (*P* < 0.05), gK (Fig. 2) (*P* < 0.05), and gL (Fig. 2) (*P* < 0.001), whereas CD86 expression was upregulated in cells transfected with gB, gC, gE, gG, gH, and gI (Fig. 2) (*P* > 0.05).

**FIG 3:**
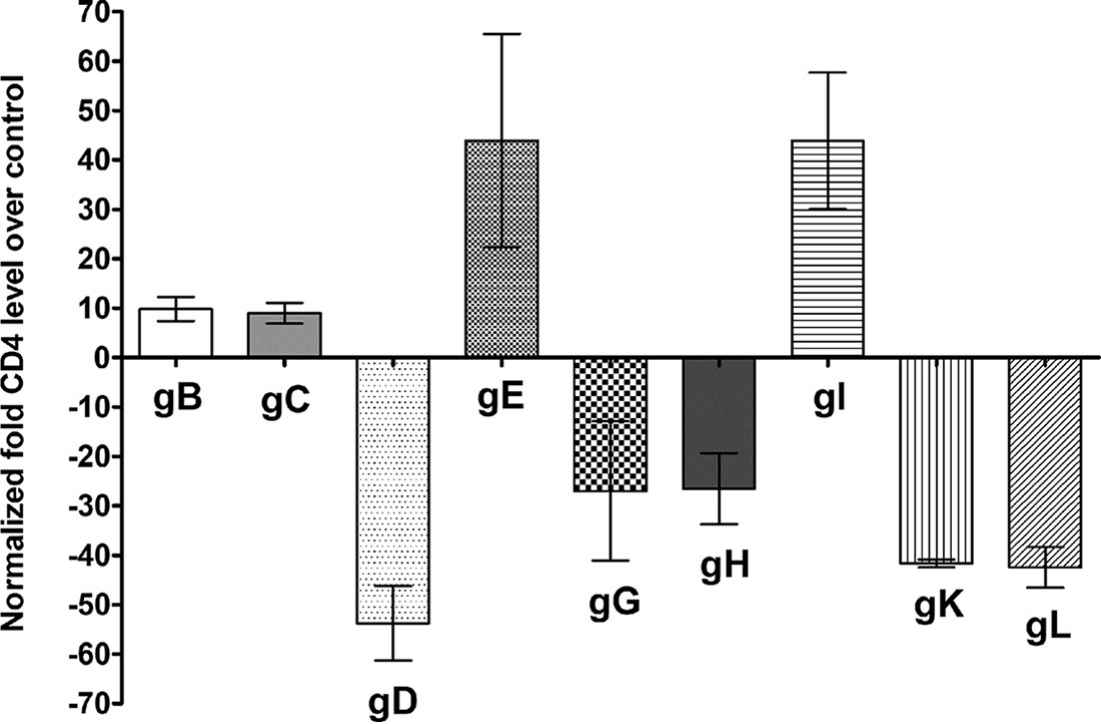
Effects of HSV-1 glycoproteins on CD4 promoter activity. 293 cells were transfected with either pGL4-EV or pGL4-CD4p DNA, and then individual plasmids expressing gB, gC, gD, gE, gG, gH, gI, gK, or gL DNA were cotransfected with plasmid DNAs. The effect of each glycoprotein on CD4 promoter activity was determined at 48 h posttransfection as we described in Materials and Methods. Assays were conducted in replicates of 10, and means ± SEM were calculated from 3 separate experiments (*n* = 30) for each point. gB is significantly upregulated compared with gD (*P* = 0.007); gC is significantly upregulated compared with gD (*P* = 0.008); gD is significantly downregulated compared with gE and gI (*P* < 0.0001); gE is significantly upregulated compared with gG, gH, gK, and gL (*P* < 0.002); gG is significantly downregulated compared with gI (*P* = 0.0007); gH is significantly downregulated compared with gI (*P* = 0.0008); and gI is significantly upregulated compared with gK and gL (*P* < 0.002). All *P* values were determined using ANOVA statistical analyses.

We next transfected 293 cells with pGL4-CD4p or pGL4-EV DNA and then individually transfected cells with gB, gC, gD, gE, gG, gH, gI, gK, and gL for 48 h. Transfected cells were harvested, and luciferase activity was determined as above. The results showed that CD4 expression was upregulated in gB- and gC-transfected groups but was significantly upregulated in cells transfected with the gE and gI glycoproteins (Fig. 3) (*P* < 0.05). Furthermore, as shown in Fig. 3, cells transfected with gD, gG, gH, gK, and gL showed significantly reduced CD4 expression.

**FIG 4:**
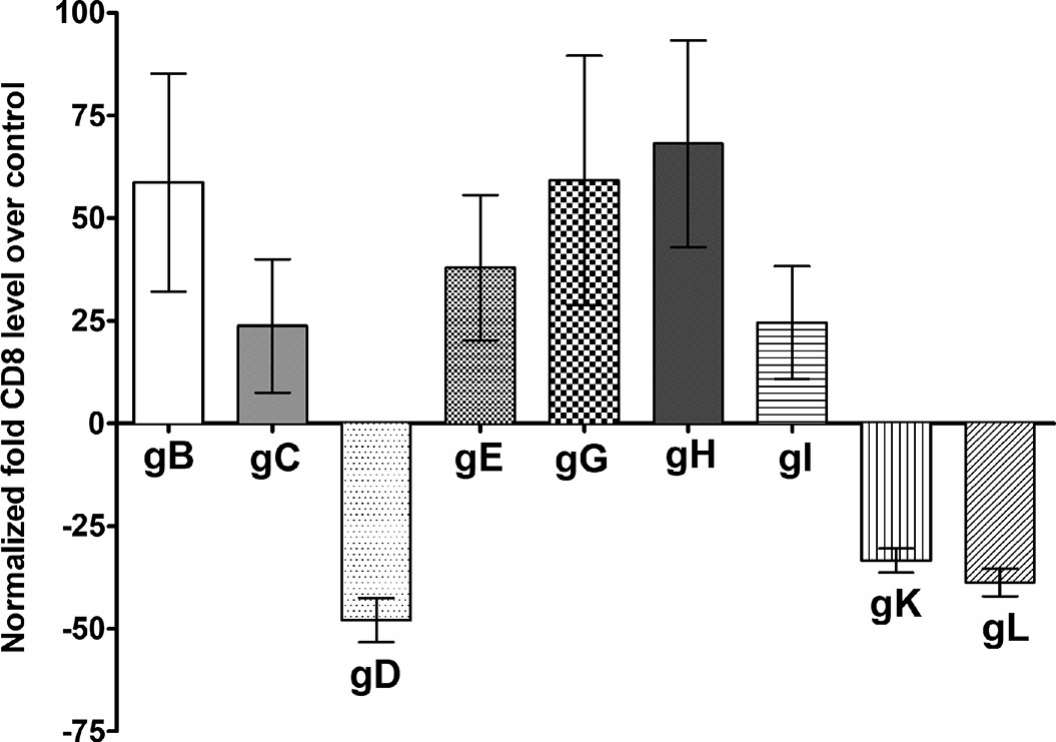
Effects of HSV-1 glycoproteins on CD8 promoter activity. 293 cells were transfected with either pGL4-EV or pGL4-CD8p DNA and then were transfected individually with gB, gC, gD, gE, gG, gH, gI, gK, and gL plasmid DNA. The effect of each glycoprotein on CD8 promoter activity was determined 48 h posttransfection as we described in Materials and Methods. Assays were conducted in replicates of 10, and means ± SEM were calculated from 3 separate experiments (*n* = 30) for each point. gC is significantly upregulated compared with gD (*P* = 0.02), and gD is significantly downregulated compared with gG and gH (*P* < 0.01). All *P* values were determined using ANOVA statistical analyses.

Lastly, the effect of the above glycoproteins on CD8 promoter activity was determined by transfecting 293 cells with pGL4-CD8p or pGL4-EV as a control, followed by transfecting cells individually with gB, gC, gD, gE, gG, gH, gI, gK, and gL plasmid DNAs. At 48 h later, the luciferase activity of each transfected group was determined as we described above and in the Materials and Methods. Similar to CD80 and CD86, CD8 promoter activity was reduced in cells expressing gD, gK, and gL (Fig. 4), while it was increased in cells expressing gB, gC, gE, gG, gH, and gI.

Based on these results, gD, gK, and gL appeared to downregulate CD80, CD86, CD4, and CD8 expression by reducing their promoter activity. Furthermore, gG and gH also appeared to uniquely reduce CD4 promoter activity. These results suggest that the assembly of a properly combined vaccine cocktail may improve the efficacy of an HSV-1 vaccine *in vivo*.

### Cytokine/chemokine expression in transfected or infected spleen cells.

The above results (Fig. 3 and 4) suggest that HSV-1 glycoproteins have distinct effects on the costimulatory and CD4 and CD8 T cell promoter activities *in vitro*. To understand what effect, if any, these individual nine glycoproteins (gB, gC, gD, gE, gG, gH, gI, gK, and gL) may have on the expression levels of cytokine/chemokines in spleen cells, we isolated total spleen cells from WT mice and transfected them with each individual glycoprotein for 48 h as described in Materials and Methods. Media from transfected cells were collected, and the expression levels of various cytokines/chemokines were analyzed by a Luminex assay (Table 1). The Luminex results revealed that interleukin-3 (IL-3) was upregulated only in cells transfected with gC, and its expression was not significantly (ns) altered in cells transfected with any of the other glycoproteins tested (Table 1). IL-9 was specifically upregulated in cells transfected with gC, gD, gH, and gL. Among the chemokines, CXCL10 (IP-10) was significantly upregulated in mouse splenocytes transfected with each of the nine glycoproteins (Table 1). The chemokine (C-C) motif ligand 2 (CCL2; MCP-1) was upregulated in splenocytes expressing each of the glycoproteins except gE, while MIP1-α was upregulated only in cells transfected with the gH, gI, gK, and gL glycoproteins (Table 1). Macrophage colony-stimulating factor (M-CSF) was upregulated in cells transfected with gD, gE, gH, and gI, but not other viral glycoproteins (Table 1).

**TABLE 1:** Cytokine/chemokine levels in spleen cells transfected with different glycoprotein genes or infected with HSV-1^*a*^

Cytokine or chemokine	Results after transfection with:									Results after infection with HSV-1	
	gB	gC	gD	gE	gG	gH	gI	gK	gL	1 PFU/cell	10 PFU/cell
GM-CSF	ns	ns	ns	↑	ns	↑	ns	ns	ns	↑	ns
IL-2	ns	ns	ns	ns	ns	ns	ns	ns	ns	ns	↑↑
IL-4	ns	ns	ns	ns	ns	ns	ns	ns	ns	↑↑↑	ns
IL-3	ns	↑↑	ns	ns	ns	ns	ns	ns	ns	ns	↑
IL-5	ns	ns	ns	↑	ns	ns	ns	ns	ns	ns	ns
IL-6	ns	ns	ns	ns	ns	ns	ns	ns	ns	↑↑	ns
IL-9	ns	↑↑↑↑	↑↑	ns	ns	↑↑	↑↑	ns	ns	ns	ns
IL-10	ns	ns	ns	ns	ns	ns	ns	ns	ns	↑↑	ns
IL-12(p40)	ns	ns	ns	ns	ns	↑	ns	ns	ns	ns	ns
IL-12(p70)	ns	ns	ns	↑	ns	ns	ns	ns	ns	ns	ns
LIF	ns	ns	ns	ns	ns	↑	↑	ns	ns	ns	ns
CXCL5 (LIX)	↑↑↑↑	ns	ns	ns	ns	ns	ns	ns	ns	↑↑	ns
IL-15	ns	ns	ns	↑	ns	↑	ns	ns	ns	ns	ns
IL-17	ns	ns	ns	ns	ns	ns	ns	ns	ns	↑	ns
CXCL10 (IP-10)	↑↑↑↑	↑↑↑↑	↑↑↑↑	↑↑	↑↑↑↑	↑↑↑↑	↑↑↑↑	↑↑↑↑	↑↑	↑↑↑	↑↑
CXCL1 (KC)	↑	ns	↑	ns	ns	ns	ns	ns	ns	↑↑	↑
CCL11 (eotaxin)	ns	ns	ns	ns	ns	ns	ns	ns	ns	↑↑	ns
CCL2 (MCP-1)	↑	↑↑↑	↑	ns	↑↑	↑↑	↑↑	↑↑↑	↑↑	↑	ns
CCL3 (MIP-1α)	ns	ns	ns	ns	ns	↑↑	↑	↑↑↑	↑↑	↑↑↑	↑
CCL4 (MIP-1β)	ns	ns	ns	ns	ns	↑	ns	ns	ns	↑↑↑	↑
M-CSF	ns	ns	↑	↑↑	ns	↑	↑	ns	ns	↑↑↑↑	↑↑
CXCL2 (MIP-2)	↑	ns	ns	ns	ns	ns	ns	ns	ns	↑↑	↑
CXCL9 (MIG)	ns	ns	ns	ns	↑	ns	ns	ns	ns	ns	ns
CCL5 (RANTES)	↑	ns	ns	ns	ns	ns	ns	ns	ns	↑↑	ns
VEGF	ns	ns	ns	ns	ns	ns	ns	ns	ns	↑	ns
TNF-α	ns	ns	ns	ns	ns	ns	ns	ns	ns	↑↑	ns

a Cytokine/chemokine levels in culture media were analyzed using mouse 32-plex panels and are shown as significant relative to vector-transfected cells. Experimental procedures are described in Materials and Methods. Briefly, isolated spleen cells from naive mice were transfected with each glycoprotein plasmid or infected with 1 or 10 PFU/cell of HSV-1 strain McKrae. Transfected or infected cell supernatants were collected. Levels of G-CSF, IFN-γ, IL-1α, IL-1β, IL-7, and IL-13 did not differ between control and transfected or infected cells and are not shown. ns, not significant relative to mock-transfected or mock-infected BMDCs. For all statistical tests, *P* values less than or equal to 0.05 were considered statistically significant and are marked by a single upward arrow (↑). Double upward arrows (↑↑) denote a *P* value of <0.01, three upward arrows (↑↑↑) equals a *P* value of <0.001, and our upward arrows (↑↑↑↑) equals *P* value of <0.0001.

Additional splenocytes were infected with 1 or 10 PFU/cell of HSV-1 strain McKrae or mock infected for 24 h. At 24 h postinfection (p.i.), media from the culture were collected and processed as above to measure the secretion of various cytokines and chemokines. Our data revealed that the majority of chemokines and cytokines were more highly expressed when splenocytes were infected with 1 PFU/cell than with 10 PFU/cell of HSV-1 strain McKrae. Of all cytokines and chemokines tested, the expressions of CXCL10 (IP-10), CXCL1 (KC), MIP-1α, MIP-1β, M-CSF, and CXCL2 (MIP-2) were significantly upregulated when infected with both 1 and 10 PFU/cell of McKrae virus for 24 h (Table 1) (*P* < 0.05). The expressions of granulocyte colony-stimulating factor (G-CSF), interferon gamma (IFN-γ), IL-1α, IL-1β, IL-7, and IL-13 were not detected in splenocytes transfected or infected in culture media. A lower expression of some cytokines/chemokines after infection with 10 PFU/cell virus could be due to complete lysis of infected cells.

### Cytokine/chemokine expression in transfected or infected BMDCs.

Bone marrow was isolated from WT mice and processed to generate BMDCs as described in Materials and Methods. Similar to splenocytes, BMDCs were first transfected with the nine viral glycoproteins listed above. Unlike CXCL10 (IP-10) which was significantly upregulated in splenocytes transfected with each glycoprotein, CXCL10 (IP-10) was not significantly expressed in BMDCs transfected with any of the glycoproteins (Table 2). IL-1β was expressed only in BMDCs transfected with gL, while IL-6 was expressed only in BMDCs transfected with gE (Table 2). Both IL-1β and IL-6 are broadly known as proinflammatory cytokines (37, 38). CCL2 (MCP-1) was significantly upregulated in BMDCs transfected with gB, gD, gE, gG, and gH, while CXCL9 (MIG) was expressed in BMDCs transfected with gE and gH (Table 2). CCL2 along with CXCL9 are chemokines which are involved in the recruitment of monocytes, memory T cells, and dendritic cells to the site of injury (39, 40). Of the various cytokine/chemokines tested, vascular endothelial growth factor (VEGF) was specifically upregulated in BMDCs transfected with gB, gD, gE, gG, and gH (Table 2).

**TABLE 2:** Cytokine/chemokine levels in BMDC transfected with different glycoprotein genes or infected with HSV-1^*a*^

Cytokine or chemokine	Results after transfection with:									Results after infection with HSV-1	
	gB	gC	gD	gE	gG	gH	gI	gK	gL	1 PFU	10 PFU
IL-1α	ns	ns	ns	ns	ns	ns	ns	ns	ns	↑↑↑	↑↑↑↑
IL-1β	ns	ns	ns	ns	ns	ns	ns	ns	↑↑	↑↑↑↑	↑↑↑↑
IL-6	ns	ns	ns	↑	ns	ns	ns	ns	ns	↑↑↑↑	ns
IL-9	ns	ns	ns	ns	ns	ns	ns	ns	ns	ns	↑↑
CXCL5 (LIX)	ns	ns	ns	ns	ns	ns	ns	ns	ns	ns	↑↑↑↑
IL-17	ns	ns	ns	ns	ns	ns	ns	ns	ns	↑↑↑↑	ns
CXCL10 (IP-10)	ns	ns	ns	ns	ns	ns	ns	ns	ns	↑↑↑↑	↑↑↑
CXCL1 (KC)	ns	ns	ns	ns	ns	ns	ns	ns	ns	↑	↑↑↑↑
CCL2 (MCP-1)	↑↑↑↑	ns	↑	↑↑	↑↑	↑↑	ns	ns	ns	↑↑↑	ns
CCL3 (MIP-1α)	ns	ns	ns	ns	ns	ns	ns	ns	ns	↑↑↑↑	↑
CCL4 (MIP-1β)	ns	ns	ns	ns	ns	ns	ns	ns	ns	↑↑↑↑	↑↑↑↑
M-CSF	ns	ns	ns	↑	ns	ns	ns	ns	ns	ns	ns
CXCL2 (MIP-2)	ns	ns	ns	ns	ns	ns	ns	ns	ns	↑↑↑	↑↑↑↑
CXCL9 (MIG)	ns	ns	ns	↑↑	↑	ns	ns	ns	ns	↑↑	ns
CCL5 (RANTES)	ns	ns	ns	ns	ns	ns	ns	ns	ns	↑↑↑	ns
VEGF	↑↑↑↑	ns	↑↑↑	↑↑↑↑	↑↑	↑↑↑↑	ns	ns	ns	ns	ns
TNF-α	ns	ns	ns	ns	ns	ns	ns	ns	ns	↑↑↑	↑↑↑

a Cytokine/chemokine levels in culture media were analyzed using mouse 32-plex panels and are shown as significant relative to vector-transfected cells. Experimental procedures are described in Materials and Methods. Briefly, BMDCs from naive mice were transfected with each glycoprotein plasmid or infected with 1 or 10 PFU/cell of HSV-1 strain McKrae. Transfected or infected cell supernatants were collected. Levels of G-CSF, eotaxin, GM-CSF, IFN-γ, IL-2, IL-4, IL-3, IL-5, IL-7, IL-10, IL-13, IL-12(p40), IL-12(p70), LIF, and IL-15 did not differ between control and transfected or infected cells and are not shown. ns, not significant relative to mock-transfected or mock-infected BMDCs. For all statistical tests, *P* values less than or equal to 0.05 were considered statistically significant and are marked by a single upward arrow (↑). Double upward arrows (↑↑) denote a *P* value of <0.01, three upward arrows (↑↑↑) equals a *P* value of <0.001, and four upward arrows (↑↑↑↑) a *P* value of <0.0001.

BMDCs were also infected with 1 or 10 PFU/cell of McKrae virus for 24 h. IL-1α, IL-1β, CXCL10 (IP-10), CXCL1 (KC), CCL3 (MIP-1α), CCL4 (MIP-1β), CXCL2 (MIP-2), and tumor necrosis factor alpha (TNF-α) were all significantly upregulated at both viral doses, while the remaining tested cytokine/chemokines were significantly upregulated only at one or the other viral dose (Table 2) (*P* < 0.05). Expression of G-CSF, eotaxin, GM-CSF, IFN-γ, IL-2, IL-4, IL-3, IL-5, IL-7, IL-10, IL-13, IL-12(p40), IL-12(p70), LIF, or IL-15 was not detected in transfected BMDCs or infected in culture media.

These results suggest that in both transfected spleens and BMDCs, and similar to promoter activities, glycoproteins affected cytokine/chemokine expression differently, thus confirming our overall hypothesis that a selective mixture of these nine glycoproteins may provide broader protection against HSV-1 infection than one or few glycoproteins.

## DISCUSSION

Both CD4^+^ T cell- and CD8^+^ T cell-mediated immune responses have been reported to be involved in protecting against ocular HSV-1 infection (41–49). The two signals required to activate T cells are mediated by CD80 and CD86, which are known as costimulatory molecules essential for T cell activation, proliferation, maintenance, and tolerance induction after binding to CD28 on T cells (17, 50). We reported recently that HSV-1 ICP22 contributes to immune escape by suppressing CD80 expression (22, 24, 25). In addition to ICP22, several HSV-1 genes, including ICP47 (51, 52), ICP0 (53, 54), gE, the gE/gI complex (55–57), and gC (58, 59) have been implicated in mechanisms of immune escape. Thus, the involvement of various HSV-1 genes in immune escape has important ramifications for the host adaptive immune response and, thus, also on the design of an effective vaccine against HSV infection. We previously constructed recombinant baculoviruses expressing high levels of each of the 11 HSV-1 glycoproteins (1–4, 12, 26–34). However, very little is known regarding the direct effect of these HSV-1 glycoproteins on CD4, CD8, CD80, and CD86 functions. Since we have shown that ICP22 suppresses CD80 (22, 24, 25) and costimulatory molecules CD80 and CD86 strengthen T cell activation and function, we extended our investigation to study the direct effect of HSV-1 glycoproteins on CD4, CD8, CD80, and CD86 expression, due to their important roles in protecting against HSV-1 infection. This information may help us design a more effective vaccine against HSV infection since many vaccines developed in mice have not performed well in human studies (36, 60).

Here, we have reported the effects of gB, gC, gD, gE, gG, gH, gI, gK, and gL on CD4, CD8, CD80, and CD86 promoter activities. After transfecting 293 cells with individual viral glycoproteins, CD4, CD8, CD80, and CD86 promoters had distinct responses to each glycoprotein. The CD80 promoter was significantly upregulated in gE-transfected cells but was not significantly affected by the expression of the other eight glycoprotein genes. Of note, gE is known to bind the Fc domain of IgG and has a role in blocking or altering the function of antibodies during cell lysis (57).

Similar to CD80, CD86 is expressed primarily on APCs, especially DCs (50, 61). CD86 promoter activity was significantly downregulated following transfection with gD and gL but was upregulated by other glycoproteins. Similar to CD80, gE upregulated CD4 activity and gI also increased CD4 activity. Although the role of gE in HSV-1 pathogenesis is not known, gE by itself (55) and the gE/gI complex have been shown to bind the Fc portion of IgG (55, 56). Binding of Fc to gE and to the gE/gI complex may help HSV-1 escape from immune cytolysis by blocking or altering the function of Fc (57). Thus, upregulation of CD80 and CD4 by gE may improve protection against HSV-1 infection. Our results showed reduced CD8 promoter activity in response to the gD, gK, and gL viral glycoproteins but upregulation of CD8 in all other tested viral glycoproteins.

Among the five glycoproteins with high neutralizing antibodies in immunized mice and protection against death in ocularly infected mice (i.e., gB, gC, gD, gE, and gI), gD is the only one that does not induce cytotoxic T lymphocyte (CTL) responses (62). CTLs play an important role in controlling HSV-1 infection (63). We have previously shown that gK plays a pathogenic role in HSV-1 infection (64). In contrast to gB, gC, gE, or gI, we found that gD did not upregulate CD4, CD8, CD80, or CD86 promoter activities, which may contribute to its lack of protection in a human vaccine study (60). We reported previously that neither gH (32) nor gL (3) induced neutralizing antibody titers in vaccinated mice and that vaccinated mice were not protected against ocular HSV-1 infection. In this study, gH upregulated CD8, CD80, and CD86 expression, while gL suppressed CD8, CD80, and CD86 expression and, together, gH and gL suppressed CD4 expression. gH and gL bind to each other, and the cell surface expression of gH requires that it be coexpressed with gL as a hetero-oligomer (65). In contrast to the lack of protection in mice following immunization with individual gH or gL (3, 32), mice vaccinated with a cocktail of gH-gL did induce neutralizing antibody titers and were protected from lethal challenge. Thus, the absence of protection with individual gH or gL could be due to their suppressive effect or because the formation of a complex between gH and gL is required to induce neutralizing antibody titers and protection in immunized mice.

Following foreign antigen stimulation, CD4^+^ and CD8^+^ T cell clones produce specific cytokine expression patterns in both mice and humans (66, 67). Based on the cytokines produced, CD4^+^ T cells are designated T_H_1 or T_H_2 and CD8**^+^** T cells are designated T_C_1 or T_C_2 (66, 68, 69). Usually, either a T_H_1/T_C_1 or a T_H_2/T_C_2 cytokine pattern predominates in response to a specific antigenic challenge (70–72). Studies have shown that viral glycoproteins have a role in activating the NF-κB pathway leading to an inflammatory cascade of cytokines/chemokines especially in the case of HSV, where gD-null virions and soluble forms of gH/gL were sufficient to elicit NF-κB activation (73). Therefore, we measured the inflammatory response to the individual viral glycoproteins when transfected or infected with HSV-1 McKrae. Based on our Luminex data, HSV-1-infected BMDCs had a higher proinflammatory response, with a significant upregulation of IL-1α, IL-1β, IL-6, IL-17, and TNF-α cytokines and chemokines, such as MCP-1, MIP-1α, MIP-1β, MIP-2, and CXCL9, than splenocytes infected with HSV-1 McKrae. These cytokines are well known for their proinflammatory nature (74).

Protection against eye disease and death is much easier to achieve than protection against virus replication and establishment of latency during HSV-1 infection. Thus, this study provides new evidence supporting a strategy to maximize vaccine efficacy by combining a selective cocktail of HSV-1 genes designed to eliminate HSV-1-induced eye disease and death in ocularly infected mice. Finally, our promoter activation results could be further mined to identify common transcription factors or pathways that could lead to specific glycoprotein responsiveness; in other words, they could be used to regulate the immune responses to achieve therapeutic treatment in the absence of viral infection.

## MATERIALS AND METHODS

### Ethics statement.

All animal procedures were performed in strict accordance with the Association for Research in Vision and Ophthalmology Statement for the Use of Animals in Ophthalmic and Vision Research and the NIH Guide for the Care and Use of Laboratory Animals (ISBN 0-309-05377-3). The animal research protocol was approved by the Institutional Animal Care and Use Committees of Cedars-Sinai Medical Center (protocol number 8837).

### Virus and cells.

Triple plaque purified WT McKrae was used in this study as we described previously (75). Rabbit skin cells (used to prepare virus stocks and determine growth kinetics) were grown in Eagle’s minimal essential media (EMEM) supplemented with 5% fetal calf serum (FCS). Cells were passaged typically at 80% confluence and grown in a 37°C incubator with 5% CO_2_. Transfection studies were conducted using HEK 293 cells (ATCC) cultured in EMEM supplemented with 10% fetal bovine serum (FBS). These cells are widely used for transfection study because of their reliable growth and great transfection efficiency potential (76). Throughout this study, HEK 293 cells are referred to as 293 cells (24). Mice used in this study were male and female 6-week-old inbred C57BL/6 (The Jackson Laboratory, Bar Harbor, ME). C57BL/6 mice were used as the source of bone marrow (BM) and splenocytes. BM cells were used to generate mouse DCs (BMDCs) as we described previously (77). Single-cell suspensions of spleen cells from individual mice were prepared as we described previously (2).

### Plasmids.

The complete open reading frame (ORF) for each of the nine HSV-1 glycoproteins (gB, gC, gD, gE, gG, gH, gI, gK, and gL) was cloned into the pVR-1055 expression vector as we described previously (9). The pVR-1055 empty vector (EV) was used as negative control. The *CD80* promoter (759 bp) (78), *CD86* promoter (700 bp) (24), *CD4* promoter (498 bp), and *CD8* promoter (468 bp) were synthesized (GenScript, Piscataway, NJ) and inserted into pGL4 multiple cloning sites to drive the expression of the luciferase reporter under each of these specific promoters. We refer to these plasmids as pGL4-CD80p, pGL4-CD86p, pGL4-CD4p, and pGL4-CD8p (Table S1).

### Transfection.

Transfection experiments were conducted using 293 cells and Gene Porter 2 (Genlantis, San Diego, CA) as we described previously (22, 24). Briefly, 293 cells were grown to 70% to 80% confluence in 12-well plates. Immediately before the experiment, plasmids were diluted in the dilution buffer provided, and transfection reagents were resuspended in EMEM media (no FBS) in individual tubes. The reagents were then combined, incubated for 5 min, and added to the plates. Cells were transfected with either promoter-less luciferase plasmid pGL4-EV or luciferase reporter plasmids driven by the CD promoters described above, namely, pGL4-CD80p, pGL4-CD86p, pGL4-CD4p, or pGL4-CD8p. pRL-SV40 (Promega, Madison, WI; catalog [cat.] E2231), a *Renilla* luciferase reporter plasmid, was used as a cotransfected internal control to monitor baseline cell responses to transfection (10 ng/reaction). By using a dual luciferase reporter system (Promega), each luciferase plasmid was transfected simultaneously with the *Renilla* control plasmid to determine responses within the same cells. Samples were prepared as described by the manufacturer (Promega). Cells were washed with phosphate-buffered saline (PBS) and lysed in lysis buffer, and the collected supernatants were transferred to 96-well plates. The luminometer (Promega; Glomax) was primed with luciferase and Stop & Glow reagents. Assays were conducted in replicates of 10, and means ± SEM were calculated from 3 separate experiments (*n* = 30).

### Luminex xMAP immunoassay.

BMDCs and spleen cells were infected with 1 or 10 PFU/cell of HSV-1 strain McKrae or mock infected for 24 h. At 24 h postinfection (p.i.), the medium was collected from infected cells and Luminex assays were performed in the Immune Assessment Core at the University of California, Los Angeles (UCLA; CA) using mouse 32-Plex magnetic cytokine/chemokine kits (EMD Millipore, Billerica, MA) according to the manufacturer’s instructions as we described previously (79). Fluorescence was quantified using a Luminex 200 instrument (Luminex Corp., Austin, TX).

### Statistical analysis.

Data were analyzed by the Student’s *t* test, one-way analysis of variance (ANOVA), or two-way ANOVA using Prism software (GraphPad, San Diego, CA). Multiple comparison tests were performed using Bonferroni analysis in GraphPad. Results were considered statistically significant if the *P* value was <0.05.

## References

[1] Ghiasi H, Slanina S, Nesburn AB, Wechsler SL. 1994. Characterization of baculovirus-expressed herpes simplex virus type 1 glycoprotein K. J Virol 68:2347–2354. doi:10.1128/jvi.68.4.2347-2354.1994. 8139020 PMC236711

[2] Ghiasi H, Kaiwar R, Nesburn AB, Slanina S, Wechsler SL. 1994. Expression of seven herpes simplex virus type 1 glycoproteins (gB, gC, gD, gE, gG, gH, and gI): comparative protection against lethal challenge in mice. J Virol 68:2118–2126. doi:10.1128/JVI.68.4.2118-2126.1994. 8138996 PMC236686

[3] Ghiasi H, Kaiwar R, Slanina S, Nesburn AB, Wechsler SL. 1994. Expression and characterization of baculovirus expressed herpes simplex virus type 1 glycoprotein L. Arch Virol 138:199–212. doi:10.1007/BF01379126. 7998829

[4] Ghiasi H, Nesburn AB, Cai S, Wechsler SL. 1998. The US5 open reading frame of herpes simplex virus type 1 does encode a glycoprotein (gJ). Intervirology 41:91–97. doi:10.1159/000024919. 9820842

[5] McGeoch DJ, Dalrymple MA, Davison AJ, Dolan A, Frame MC, McNab D, Perry LJ, Scott JE, Taylor P. 1988. The complete DNA sequence of the long unique region in the genome of herpes simplex virus type 1. J Gen Virol 69:1531–1574. doi:10.1099/0022-1317-69-7-1531. 2839594

[6] Roizman B, Keller JM, Spear PG, Terni M, Nahmias A, Dowdle W. 1970. Variability, structural glycoproteins, and classification of herpes simplex viruses. Nature 227:1253–1254. doi:10.1038/2271253a0. 4318128

[7] Spear PG. 1976. Membrane proteins specified by herpes simplex viruses. I. Identification of four glycoprotein precursors and their products in type 1-infected cells. J Virol 17:991–1008. doi:10.1128/JVI.17.3.991-1008.1976. 176453 PMC515499

[8] Stannard LM, Fuller AO, Spear PG. 1987. Herpes simplex virus glycoproteins associated with different morphological entities projecting from the virion envelope. J Gen Virol 68:715–725. doi:10.1099/0022-1317-68-3-715. 3029300

[9] Osorio Y, Cohen J, Ghiasi H. 2004. Improved protection from primary ocular HSV-1 infection and establishment of latency using multigenic DNA vaccines. Invest Ophthalmol Vis Sci 45:506–514. doi:10.1167/iovs.03-0828. 14744892

[10] Spear PG, Sarmiento M, Manservigi R. 1978. The structural proteins and glycoproteins of herpesviruses: a review. IARC Sci Publ 24:157–167. 376432

[11] Jennings SR, Lippe PA, Pauza KJ, Spear PG, Pereira L, Tevethia SS. 1987. Kinetics of expression of herpes simplex virus type 1-specific glycoprotein species on the surfaces of infected murine, simian, and human cells: flow cytometric analysis. J Virol 61:104–112. doi:10.1128/JVI.61.1.104-112.1987. 3023688 PMC255213

[12] Ghiasi H, Bahri S, Nesburn AB, Wechsler SL. 1995. Protection against herpes simplex virus-induced eye disease after vaccination with seven individually expressed herpes simplex virus 1 glycoproteins. Invest Ophthalmol Vis Sci 36:1352–1360. 7775113

[13] Burke RL. 1991. Development of a herpes simplex virus subunit glycoprotein vaccine for prophylactic and therapeutic use. Rev Infect Dis 13:S906–S911. doi:10.1093/clind/13.Supplement_11.S906. 1664126

[14] Burke RL. 1992. Contemporary approaches to vaccination against herpes simplex virus. Curr Top Microbiol Immunol 179:137–158. doi:10.1007/978-3-642-77247-4_9. 1499347

[15] Toka FN, Suvas S, Rouse BT. 2004. CD4+ CD25+ T cells regulate vaccine-generated primary and memory CD8+ T-cell responses against herpes simplex virus type 1. J Virol 78:13082–13089. doi:10.1128/JVI.78.23.13082-13089.2004. 15542660 PMC525021

[16] Gimenez F, Suryawanshi A, Rouse BT. 2013. Pathogenesis of herpes stromal keratitis—a focus on corneal neovascularization. Prog Retin Eye Res 33:1–9. doi:10.1016/j.preteyeres.2012.07.002. 22892644 PMC3511644

[17] Greenfield EA, Nguyen KA, Kuchroo VK. 1998. CD28/B7 costimulation: a review. Crit Rev Immunol 18:389–418. doi:10.1615/critrevimmunol.v18.i5.10. 9784967

[18] Lenschow DJ, Su GH, Zuckerman LA, Nabavi N, Jellis CL, Gray GS, Miller J, Bluestone JA. 1993. Expression and functional significance of an additional ligand for CTLA-4. Proc Natl Acad Sci USA 90:11054–11058. doi:10.1073/pnas.90.23.11054. 7504292 PMC47920

[19] Hathcock KS, Laszlo G, Pucillo C, Linsley P, Hodes RJ. 1994. Comparative analysis of B7-1 and B7-2 costimulatory ligands: expression and function. J Exp Med 180:631–640. doi:10.1084/jem.180.2.631. 7519245 PMC2191623

[20] Inaba K, Witmer-Pack M, Inaba M, Hathcock KS, Sakuta H, Azuma M, Yagita H, Okumura K, Linsley PS, Ikehara S, Muramatsu S, Hodes RJ, Steinman RM. 1994. The tissue distribution of the B7-2 costimulator in mice: abundant expression on dendritic cells in situ and during maturation in vitro. J Exp Med 180:1849–1860. doi:10.1084/jem.180.5.1849. 7525841 PMC2191729

[21] Larsen CP, Ritchie SC, Hendrix R, Linsley PS, Hathcock KS, Hodes RJ, Lowry RP, Pearson TC. 1994. Regulation of immunostimulatory function and costimulatory molecule (B7-1 and B7-2) expression on murine dendritic cells. J Immunol 152:5208–5219. 7514631

[22] Matundan HH, Wang S, Jaggi U, Yu J, Ghiasi H. 2021. Suppression of CD80 expression by ICP22 affects herpes simplex virus type 1 replication and CD8(+)IFN-gamma(+) infiltrates in the eyes of infected mice but not latency reactivation. J Virol 95:e0103621. doi:10.1128/JVI.01036-21. 34287036 PMC8428405

[23] Lim TS, Goh JK, Mortellaro A, Lim CT, Hammerling GJ, Ricciardi-Castagnoli P. 2012. CD80 and CD86 differentially regulate mechanical interactions of T-cells with antigen-presenting dendritic cells and B-cells. PLoS One 7:e45185. doi:10.1371/journal.pone.0045185. 23024807 PMC3443229

[24] Matundan H, Ghiasi H. 2019. Herpes simplex virus 1 ICP22 suppresses CD80 expression by murine dendritic cells. J Virol 93:e01803-18. doi:10.1128/JVI.01803-18. 30404803 PMC6340034

[25] Matundan HH, Jaggi U, Wang S, Ghiasi H. 2019. Loss of ICP22 in HSV-1 elicits immune infiltration and maintains stromal keratitis despite reduced primary and latent virus infectivity. Invest Ophthalmol Vis Sci 60:3398–3406. doi:10.1167/iovs.19-27701. 31387116 PMC6685448

[26] Ghiasi H, Nesburn AB, Kaiwar R, Wechsler SL. 1991. Immunoselection of recombinant baculoviruses expressing high levels of biologically active herpes simplex virus type 1 glycoprotein D. Arch Virol 121:163–178. doi:10.1007/BF01316752. 1662037

[27] Ghiasi H, Kaiwar R, Nesburn AB, Slanina S, Wechsler SL. 1992. Baculovirus-expressed glycoprotein E (gE) of herpes simplex virus type-1 (HSV-1) protects mice against lethal intraperitoneal and lethal ocular HSV-1 challenge. Virology 188:469–476. doi:10.1016/0042-6822(92)90500-o. 1585630

[28] Ghiasi H, Kaiwar R, Nesburn AB, Wechsler SL. 1992. Baculovirus expressed herpes simplex virus type 1 glycoprotein C protects mice from lethal HSV-1 infection. Antiviral Res 18:291–302. doi:10.1016/0166-3542(92)90062-a. 1416910

[29] Ghiasi H, Kaiwar R, Nesburn AB, Wechsler SL. 1992. Expression of herpes simplex virus type 1 glycoprotein I in baculovirus: preliminary biochemical characterization and protection studies. J Virol 66:2505–2509. doi:10.1128/JVI.66.4.2505-2509.1992. 1548774 PMC289047

[30] Ghiasi H, Kaiwar R, Nesburn AB, Wechsler SL. 1992. Expression of herpes simplex virus type 1 glycoprotein B in insect cells. Initial analysis of its biochemical and immunological properties. Virus Res 22:25–39. doi:10.1016/0168-1702(92)90087-p. 1311136

[31] Ghiasi H, Kaiwar R, Nesburn AB, Wechsler SL. 1992. Baculovirus-expressed glycoprotein G of herpes simplex virus type 1 partially protects vaccinated mice against lethal HSV-1 challenge. Virology 190:233–239. doi:10.1016/0042-6822(92)91209-d. 1529531

[32] Ghiasi H, Kaiwar R, Nesburn AB, Wechsler SL. 1992. Baculovirus-expressed glycoprotein H of herpes simplex virus type 1 (HSV-1) induces neutralizing antibody and delayed type hypersensitivity responses, but does not protect immunized mice against lethal HSV-1 challenge. J Gen Virol 73:719–722. doi:10.1099/0022-1317-73-3-719. 1312127

[33] Ghiasi H, Cai S, Slanina S, Nesburn AB, Wechsler SL. 1997. Nonneutralizing antibody against the glycoprotein K of herpes simplex virus type-1 exacerbates herpes simplex virus type-1-induced corneal scarring in various virus-mouse strain combinations. Invest Ophthalmol Vis Sci 38:1213–1221. 9152241

[34] Ghiasi H, Cai S, Nesburn AB, Wechsler SL. 1996. Vaccination with herpes simplex virus type 1 glycoprotein K impairs clearance of virus from the trigeminal ganglia resulting in chronic infection. Virology 224:330–333. doi:10.1006/viro.1996.0537. 8862430

[35] Ghiasi H, Nesburn AB, Wechsler SL. 1996. Vaccination with a cocktail of seven recombinantly expressed HSV-1 glycoproteins protects against ocular HSV-1 challenge more efficiently than vaccination with any individual glycoprotein. Vaccine 14:107–112. doi:10.1016/0264-410x(95)00169-2. 8852405

[36] Corey L, Langenberg AG, Ashley R, Sekulovich RE, Izu AE, Douglas JM, Jr, Handsfield HH, Warren T, Marr L, Tyring S, DiCarlo R, Adimora AA, Leone P, Dekker CL, Burke RL, Leong WP, Straus SE. 1999. Recombinant glycoprotein vaccine for the prevention of genital HSV-2 infection: two randomized controlled trials. JAMA 282:331–340. doi:10.1001/jama.282.4.331. 10432030

[37] Dinarello CA. 1996. Biologic basis for interleukin-1 in disease. Blood 87:2095–2147. doi:10.1182/blood.V87.6.2095.bloodjournal8762095. 8630372

[38] Tanaka T, Narazaki M, Kishimoto T. 2014. IL-6 in inflammation, immunity, and disease. Cold Spring Harb Perspect Biol 6:a016295. doi:10.1101/cshperspect.a016295. 25190079 PMC4176007

[39] Carr MW, Roth SJ, Luther E, Rose SS, Springer TA. 1994. Monocyte chemoattractant protein 1 acts as a T-lymphocyte chemoattractant. Proc Natl Acad Sci USA 91:3652–3656. doi:10.1073/pnas.91.9.3652. 8170963 PMC43639

[40] Tokunaga R, Zhang W, Naseem M, Puccini A, Berger MD, Soni S, McSkane M, Baba H, Lenz HJ. 2018. CXCL9, CXCL10, CXCL11/CXCR3 axis for immune activation—a target for novel cancer therapy. Cancer Treat Rev 63:40–47. doi:10.1016/j.ctrv.2017.11.007. 29207310 PMC5801162

[41] Ghiasi H, Cai S, Perng GC, Nesburn AB, Wechsler SL. 2000. Both CD4+ and CD8+ T cells are involved in protection against HSV-1 induced corneal scarring. Br J Ophthalmol 84:408–412. doi:10.1136/bjo.84.4.408. 10729300 PMC1723442

[42] Erlich KS, Wofsy D, Dix RD, Mills J. 1989. Effects of selective depletion of L3T4+ T-lymphocytes on herpes simplex virus encephalitis. Clin Immunol Immunopathol 52:190–201. doi:10.1016/0090-1229(89)90171-2. 2525439

[43] Oakes JE, Rector JT, Lausch RN. 1984. Lyt-1+ T cells participate in recovery from ocular herpes simplex virus type 1 infection. Invest Ophthalmol Vis Sci 25:188–194. 6321384

[44] Hendricks RL, Tumpey TM. 1990. Contribution of virus and immune factors to herpes simplex virus type I- induced corneal pathology. Invest Ophthalmol Vis Sci 31:1929–1939. 2170289

[45] Staats HF, Oakes JE, Lausch RN. 1991. Anti-glycoprotein D monoclonal antibody protects against herpes simplex virus type 1-induced diseases in mice functionally depleted of selected T-cell subsets or asialo GM1+ cells. J Virol 65:6008–6014. doi:10.1128/JVI.65.11.6008-6014.1991. 1920624 PMC250266

[46] Manickan E, Rouse BT. 1995. Roles of different T-cell subsets in control of herpes simplex virus infection determined by using T-cell-deficient mouse-models. J Virol 69:8178–8179. doi:10.1128/JVI.69.12.8178-8179.1995. 7494346 PMC189778

[47] Manickan E, Francotte M, Kuklin N, Dewerchin M, Molitor C, Gheysen D, Slaoui M, Rouse BT. 1995. Vaccination with recombinant vaccinia viruses expressing ICP27 induces protective immunity against herpes simplex virus through CD4+ Th1+ T cells. J Virol 69:4711–4716. doi:10.1128/JVI.69.8.4711-4716.1995. 7609036 PMC189277

[48] Newell CK, Martin S, Sendele D, Mercadal CM, Rouse BT. 1989. Herpes simplex virus-induced stromal keratitis: role of T-lymphocyte subsets in immunopathology. J Virol 63:769–775. doi:10.1128/JVI.63.2.769-775.1989. 2536102 PMC247749

[49] Nash AA, Jayasuriya A, Phelan J, Cobbold SP, Waldmann H, Prospero T. 1987. Different roles for L3T4+ and Lyt 2+ T cell subsets in the control of an acute herpes simplex virus infection of the skin and nervous system. J Gen Virol 68:825–833. doi:10.1099/0022-1317-68-3-825. 2950204

[50] Sharpe AH, Freeman GJ. 2002. The B7-CD28 superfamily. Nat Rev Immunol 2:116–126. doi:10.1038/nri727. 11910893

[51] Hill A, Jugovic P, York I, Russ G, Bennink J, Yewdell J, Ploegh H, Johnson D. 1995. Herpes simplex virus turns off the TAP to evade host immunity. Nature 375:411–415. doi:10.1038/375411a0. 7760935

[52] Goldsmith K, Chen W, Johnson DC, Hendricks RL. 1998. Infected cell protein (ICP)47 enhances herpes simplex virus neurovirulence by blocking the CD8+ T cell response. J Exp Med 187:341–348. doi:10.1084/jem.187.3.341. 9449714 PMC2212130

[53] Heilingloh CS, Muhl-Zurbes P, Steinkasserer A, Kummer M. 2014. Herpes simplex virus type 1 ICP0 induces CD83 degradation in mature dendritic cells independent of its E3 ubiquitin ligase function. J Gen Virol 95:1366–1375. doi:10.1099/vir.0.062810-0. 24643878

[54] van Lint AL, Murawski MR, Goodbody RE, Severa M, Fitzgerald KA, Finberg RW, Knipe DM, Kurt-Jones EA. 2010. Herpes simplex virus immediate-early ICP0 protein inhibits Toll-like receptor 2-dependent inflammatory responses and NF-kappaB signaling. J Virol 84:10802–10811. doi:10.1128/JVI.00063-10. 20686034 PMC2950559

[55] Bell S, Cranage M, Borysiewicz L, Minson T. 1990. Induction of immunoglobulin G Fc receptors by recombinant vaccinia viruses expressing glycoproteins E and I of herpes simplex virus type 1. J Virol 64:2181–2186. doi:10.1128/JVI.64.5.2181-2186.1990. 2157879 PMC249377

[56] Johnson DC, Frame MC, Ligas MW, Cross AM, Stow ND. 1988. Herpes simplex virus immunoglobulin G Fc receptor activity depends on a complex of two viral glycoproteins, gE and gI. J Virol 62:1347–1354. doi:10.1128/JVI.62.4.1347-1354.1988. 2831396 PMC253147

[57] Adler R, Glorioso JC, Cossman J, Levine M. 1978. Possible role of Fc receptors on cells infected and transformed by herpesvirus: escape from immune cytolysis. Infect Immun 21:442–447. doi:10.1128/iai.21.2.442-447.1978. 80379 PMC422016

[58] Eisenberg RJ, Ponce de Leon M, Friedman HM, Fries LF, Frank MM, Hastings JC, Cohen GH. 1987. Complement component C3b binds directly to purified glycoprotein C of herpes simplex virus types 1 and 2. Microb Pathog 3:423–435. doi:10.1016/0882-4010(87)90012-x. 2849025

[59] Friedman HM, Wang L, Fishman NO, Lambris JD, Eisenberg RJ, Cohen GH, Lubinski J. 1996. Immune evasion properties of herpes simplex virus type 1 glycoprotein gC. J Virol 70:4253–4260. doi:10.1128/JVI.70.7.4253-4260.1996. 8676446 PMC190356

[60] Belshe RB, Leone PA, Bernstein DI, Wald A, Levin MJ, Stapleton JT, Gorfinkel I, Morrow RL, Ewell MG, Stokes-Riner A, Dubin G, Heineman TC, Schulte JM, Deal CD, Herpevac Trial for Women. 2012. Efficacy results of a trial of a herpes simplex vaccine. N Engl J Med 366:34–43. doi:10.1056/NEJMoa1103151. 22216840 PMC3287348

[61] Lenschow DJ, Walunas TL, Bluestone JA. 1996. CD28/B7 system of T cell costimulation. Annu Rev Immunol 14:233–258. doi:10.1146/annurev.immunol.14.1.233. 8717514

[62] Ghiasi H, Nesburn AB, Wechsler SL. 1991. Cell surface expression of herpes simplex virus type 1 glycoprotein H in recombinant baculovirus-infected cells. Virology 185:187–194. doi:10.1016/0042-6822(91)90766-5. 1656584

[63] Martin S, Cantin E, Rouse BT. 1988. Cytotoxic T lymphocytes. Their relevance in herpesvirus infections. Ann N Y Acad Sci 532:257–272. doi:10.1111/j.1749-6632.1988.tb36344.x. 2845846

[64] Jaggi U, Wang S, Tormanen K, Matundan H, Ljubimov AV, Ghiasi H. 2018. Role of herpes simplex virus type 1 (HSV-1) glycoprotein K (gK) pathogenic CD8(+) T cells in exacerbation of eye disease. Front Immunol 9:2895. doi:10.3389/fimmu.2018.02895. 30581441 PMC6292954

[65] Hutchinson L, Browne H, Wargent V, Davis-Poynter N, Primorac S, Goldsmith K, Minson AC, Johnson DC. 1992. A novel herpes simplex virus glycoprotein, gL, forms a complex with glycoprotein H (gH) and affects normal folding and surface expression of gH. J Virol 66:2240–2250. doi:10.1128/JVI.66.4.2240-2250.1992. 1312629 PMC289017

[66] Mosmann TR, Cherwinski H, Bond MW, Giedlin MA, Coffman RL. 1986. Two types of murine helper T cell clone. I. Definition according to profiles of lymphokine activities and secreted proteins. J Immunol 136:2348–2357. 2419430

[67] Mosmann TR, Coffman RL. 1989. TH1 and TH2 cells: different patterns of lymphokine secretion lead to different functional properties. Annu Rev Immunol 7:145–173. doi:10.1146/annurev.iy.07.040189.001045. 2523712

[68] Abbas AK, Murphy KM, Sher A. 1996. Functional diversity of helper T lymphocytes. Nature 383:787–793. doi:10.1038/383787a0. 8893001

[69] Li L, Sad S, Kagi D, Mosmann TR. 1997. CD8Tc1 and Tc2 cells secrete distinct cytokine patterns in vitro and in vivo but induce similar inflammatory reactions. J Immunol 158:4152–4161. 9126975

[70] Bendelac A, Schwartz RH. 1991. CD4+ and CD8+ T cells acquire specific lymphokine secretion potentials during thymic maturation. Nature 353:68–71. doi:10.1038/353068a0. 1831881

[71] Ghiasi H, Wechsler SL, Kaiwar R, Nesburn AB, Hofman FM. 1995. Local expression of tumor necrosis factor alpha and interleukin-2 correlates with protection against corneal scarring after ocular challenge of vaccinated mice with herpes simplex virus type 1. J Virol 69:334–340. doi:10.1128/JVI.69.1.334-340.1995. 7983727 PMC188580

[72] Biron CA. 1994. Cytokines in the generation of immune responses to, and resolution of, virus infection. Curr Opin Immunol 6:530–538. doi:10.1016/0952-7915(94)90137-6. 7946039

[73] Leoni V, Gianni T, Salvioli S, Campadelli-Fiume G. 2012. Herpes simplex virus glycoproteins gH/gL and gB bind Toll-like receptor 2, and soluble gH/gL is sufficient to activate NF-kappaB. J Virol 86:6555–6562. doi:10.1128/JVI.00295-12. 22496225 PMC3393584

[74] Kumaraguru U, Davis I, Rouse BT. 1999. Chemokines and ocular pathology caused by corneal infection with herpes simplex virus. J Neurovirol 5:42–47. doi:10.3109/13550289909029744. 10190689

[75] Matundan HH, Mott KR, Allen SJ, Wang S, Bresee CJ, Ghiasi YN, Town T, Wechsler SL, Ghiasi H. 2016. Interrelationship of primary virus replication, level of latency, and time to reactivation in the trigeminal ganglia of latently infected mice. J Virol 90:9533–9542. doi:10.1128/JVI.01373-16. 27512072 PMC5044812

[76] Swiech K, Kamen A, Ansorge S, Durocher Y, Picanco-Castro V, Russo-Carbolante EM, Neto MS, Covas DT. 2011. Transient transfection of serum-free suspension HEK 293 cell culture for efficient production of human rFVIII. BMC Biotechnol 11:114. doi:10.1186/1472-6750-11-114. 22115125 PMC3254136

[77] Mott KR, Underhill D, Wechsler SL, Town T, Ghiasi H. 2009. A role for the JAK-STAT1 pathway in blocking replication of HSV-1 in dendritic cells and macrophages. Virol J 6:56. doi:10.1186/1743-422X-6-56. 19439086 PMC2686698

[78] Selvakumar A, White PC, Dupont B. 1993. Genomic organization of the mouse B-lymphocyte activation antigen B7. Immunogenetics 38:292–295. doi:10.1007/BF00188807. 7686531

[79] Jaggi U, Matundan HH, Yu J, Hirose S, Mueller M, Wormley FL, Jr, Ghiasi H. 2021. Essential role of M1 macrophages in blocking cytokine storm and pathology associated with murine HSV-1 infection. PLoS Pathog 17:e1009999. doi:10.1371/journal.ppat.1009999. 34653236 PMC8550391

