# Supplementary material for: Herpes Simplex Virus 1 Glycoproteins Differentially Regulate the Activity of Costimulatory Molecules and T Cells
Source: mSphere. 2022 Sep 12;7(5):e00382-22. doi: 10.1128/msphere.00382-22 (PMC9599263; doi:10.1128/msphere.00382-22)
Supplement: TABLE S1 [file msphere.00382-22-s0001.docx]

CD80 Promoter:

SacI (1)CTTACTTTCTTACTTTCTTTCTTTTCTGTTTTTTGGTTTTTCGAGACAGGGTTTCTT TGTATAGCCCTGGCTGTCCTGGAACTCACTCTGTAGACCAGGCTGGCCTCGAACTCAGAAATCTGCCTCTGCCTTTACCTCCTGAGTGCTGGGAATTAAAGGTGTGCACCACCATGCCCGGCTGGGATGTCATTCGTTTTCATTTCTCAATTTTGATACTTTATGGAAGAAAAAAGAAAAGATAGACAAGCCTCTTCATGTAATACTCCATAGTCTCAATAAGTGGTGTTCGTAACGTGGCTTCTCTTTCCTTACCTTTTACTGGTAGATTTCTCGGTTGATTGATGTCCCTGTAGGACTTACTGGGTTTAAGATTCTTGGTTTCCTGTTTTAAGATATAAAGAAACCATTTCCTAACTAAAACACTGCCTTGGACAAATATACTTTTGGCAGTCACTCTGTGTCCAGAATGGAATTTAAGCTTTCATGGCCTAGCTGCTAGTGAAGGTTCTCTGCTTTTTTTTGGCTGTTGTATGTGAAATGGGGTTGGGTGGGAACCACCTCACTGTGTTCTAGTGTTAGTCACCCCACCCCCGCAAGCAGAATCCTTTTACCCAGCTTTTTCACCCAGCTGTGCTCACCCGGTGCTCAGAACAGGCCTGGACAAGTCACCTCCCCTAGAGTTCTGGGGACCTTTGAGTTGCCCTCATGGCCACACCCTGATTCAGAACTCTCACTCTGTCGTAAGATAGAGCTACTGGG(759) XhoI

CD86Promoter:

BamH1 (1)GGATGTTATAGGCTTCTCCTCAGCAGGGTGGAGCAGCTCTGATGGAGGGGC

TCCAGCACTGCCTTGTAGAAGCCTCTTTATTGCTGTACGAGTCACTTTTAGCAAGTCAATTTCCGCATACCTAAACCCCTTATCTGATATAGTTGTCTGAAGGCAACAGTCACCTAAGCACTTCTCAGCCCTATAAGGCTTCCCATTTCGCCAGGTTATGTCTAAGGACTATGGTAGCTCTATCAAGTGGACAAAGTTGGTCAGTTGATTTCACAACATTGCTTGTCTTGGGCTTCTTGGTTTCTTTTGAACATCTTGTTGGAACTCAAGGCACTGAATGTTTTGATTTGTTTTGTTTTTACAGTTGGATTTTTAAAAATTGGTTATTTTCTTTATTTACATTTCAAATGTTATCCCCTGTCCCACCCACTCCTGCCTGCCCCCTGAATTGTTTAAAAAACAAACAAAACCCTCAAGACTGAAACCGAACTCGAATAATCTGCAAAGCAAAGAAAGGGTTAAAGATATCTTCCTCAAGTCTAAGAAAAGAGGAACAACCCTTAAGAAAAAGTCAACCACCAGGGTACACAGTCATTGCTGAGGAAGAAAGAGGAGCAAGCAGACGCGTAAGAGTGGCTCCTGTAGGCAGCACGGACTTGAACAACCAGACTCCTGTAGACGTGTTCCAGAACTTACGGAAGCACCCACG(700) BamH1

CD4 Promoter:

BamH1 (1)GAGCTCTCTAGAACAGCCGACTTAGTGGATCTGTTGGGCAACGTAATCTAAA

TTTTCCATTGATTAAATTGCTAACTTTTTTTTTTAAAACATGGCATTACTGCATAGGCAGCCTAGTCTGGCCTTGAGCTTGTGATTTTTCTGCCTAAATCACCCAGTGCTGGGGTTACAGTTACATGCCCGTGCCCGTGCCCGTGCCTGTAAGCCTTGCCTCACGTTGACCTAACCAGGCTGTTTCAGCTTTTTACGGCACCAAACAATAGTGACATTCCAGACTCCAGCTTGATTCTGCTCAGGCATTTTCAGTGACTTTGGCATTTTTAATTTTTCAACTTCCCCAACAACTGGGGGTGGGAGGGAGGGACTCCTGAGGGCTGGCTTACGTCCGCCGTGCAGAGGAGCCTCACGACCAGGCTTCCTGTCTTTTCATTTACGAACATCTGTGAAGGCAAAGCAAGACTCTCTTCTTCACTAGGTACCTGTTTGCAAAGTCTCGAG(498) BamHI

CD8 Promoter:

BamH1 (1)GAGCTCAAGCTTGGGGAACCCAAGAACCTATCTACAAGAAAAAGAAAAGGG

AGAGTGGGCACTCCTCTCCCTTCATGTCCTGTCACTCTCATATTTTTTTTATTAAGATTTAATCTTTATCCTTCTTCTTTCAGCCACTCTTGCAGAAGAACAATTCCTAAGCCTCCCTTCCAAGTGTTTTCTCTTCCCCAAATCTCAGTTTTCAATTCCTCCCACTTGCTCTGCAAGGGTGCATTCTCACTCTGAGTTCCCAGGCCCCTAAAAGGTGGTTGACACTCTTTGGTGGGGACTTTGGGTGACATCATATCCTCACATAGGAAATCAGCTCTGTCTGCAGCTGGCTAAAGGAGCAGTTTCCCCGACCCTACACGCCTCCCCCACCGCACCTCCTCCGCCCTGTTCCTGGGCCCCTCCCCTAGAGCCCTAGCTTGACCTAAGCTGCTTGCTGGTGGAGAGCACACCCTCGAG(468) BamHI
